# Preterm Assessment at Birth and Its Association With the Development of Retinopathy of Prematurity (ROP): A Tertiary Care Center Experience

**DOI:** 10.7759/cureus.33126

**Published:** 2022-12-30

**Authors:** Omar A Bokhary, Abdulelah G Abumohssin, Maha K Alghamdi, Suhail K Abualnaja, Hamza L Fida, Nizar M Alhibshi

**Affiliations:** 1 Medicine and Surgery, King Abdulaziz University, Jeddah, SAU; 2 Ophthalmology, King Abdulaziz University Hospital, Jeddah, SAU

**Keywords:** neonate, retina, preterm, retinopathy of prematurity, rop

## Abstract

Background: Prematurity is a leading cause of neonatal morbidity and mortality and is associated with insufficient development of multiple body structures, including neurovascular and retinal tissues. Retinopathy of prematurity (ROP) is an abnormal vaso proliferation of the neonatal retina that results from an arrest in the normal development of the retinal nerve and blood supply. Incidence has been increasing due to advancements in intensive care and survival of preterm neonates, as well as improvements in screening methods for ROP.

Objectives: The objective is to assess the initial clinical and laboratory characteristics of preterm infants at the time of birth to identify population-specific risk factors for the development of ROP in a tertiary care center in western Saudi Arabia.

Methods and materials: This was a retrospective record review conducted at King Abdulaziz University Hospital (KAUH) in Jeddah, Saudi Arabia. The study included 37 patients diagnosed with ROP. Their ROP staging, complete blood count, appearance, pulse, grimace, activity and respiration (APGAR) score, and birth characteristics were all analyzed.

Results: Thirty-seven neonates diagnosed with ROP and who met the study inclusion criteria were included. our results showed a female predominance of 51.4%, the mean age of the pregnancy was 27.18 ± 2.29 weeks, the mean birth weight was 0.8 ± 0.26, and 66.7% of our sample was delivered by the cesarean section. A significant association was found between the birth weight and the development of ROP in the right eye (p = 0.026); another significance was found between gestational age and the development of ROP in the same eye (p = 0.016).

Conclusions: A low birth weight and gestational age show a significant association with the development of ROP. Early identification and treatment of ROP are important to preserve a neonate's eyesight.

## Introduction

Preterm birth, defined as a delivery that occurs before the completion of 37 weeks of gestation, occurs more frequently than once in every 10 live births [1-3]. Prematurity is a leading cause of neonatal morbidity and mortality and is associated with insufficient development of multiple body structures, including neurovascular and retinal tissues [4,5]. Retinopathy of prematurity (ROP) is an abnormal vasoproliferation of the neonatal retina that results from an arrest in the normal development of the retinal nerve and blood supply [4]. The worldwide prevalence of ROP has been reported to be greater than 50,000 [6], and its incidence has been increasing due to advancements in neonatal intensive care and the survival of preterm neonates [7], as well as improvements in early screening programs for ROP [8]. Local studies in Saudi Arabia have confirmed the high prevalence of ROP, with a rate of 56% of premature infants [9-14]. An international classification is widely used now, which was proposed in the 1980s and modified in 2005, with the goal of standardizing the description of ROP through the classification of different stages [15]. Several risk factors for the development of ROP have been proposed in past literature. Frequently reported risk factors include oxygen supplementation, low gestational age, low birth weight, hyperglycemia, obstetric complications, and low Appearance, Pulse, Grimace, Activity and Respiration (APGAR) scores [4,16]. The risk factors most consistently reported were decreasing gestational age and birth weight [17,18], and both were similarly reported in single-center studies done in Saudi Arabia [9-11,19]. The importance of early detection and timely intervention for ROP could not be overstated, as Al Hadlaq demonstrated that late diagnosis was significantly associated with the indicated treatment [18]. Furthermore, as Saudi Arabia is experiencing advancements in both primary prenatal care and neonatal intensive care, risk factors for ROP must be thoroughly investigated in order to prevent them if possible. This study, therefore, aims to assess the initial clinical and laboratory characteristics of preterm infants at the time of birth in order to identify population-specific risk factors for the development of ROP in a tertiary care center in the western region of Saudi Arabia.

## Materials and methods

This single-center retrospective record review was conducted at King Abdulaziz University Hospital (KAUH), a tertiary care center in Jeddah, Saudi Arabia. This research was authorized by the Institutional Review Board of KAUH (Ref: 603-21). The procedures used were under the responsible committee's ethical standards based on the Good Clinical Practice Guidelines. Due to the study's retrospective nature, informed consent was waived. We collected and analyzed the data of 1,000 preterms born between January 2010 and January 2021. The number of preterms who fulfilled the inclusion criteria was 37; we included all preterms with documented follow-up visits and a diagnosis of ROP. Preterms with poor documentation, not classified according to the international classification of ROP (ICROP), and infants with congenital malformations were excluded. The clinical data of patients obtained from the medical records included demographic data such as gestational age, Gender, mode of delivery (spontaneous vaginal delivery or cesarian section), and birth weight. Furthermore, 1, 5-minute APGAR score, and Complete Blood Count values: hemoglobin level (Hb), hematocrit value (Hct), leucocyte count (WBC), mean corpuscular hemoglobin (MCH), mean corpuscular volume (MCV), red cell distribution width (RDW) and platelet count were collected.

Staging

ROP was staged for each eye individually by an experienced ophthalmologist. The right eye was referred to as oculus dextrus (OD) and the left eye as oculus sinister (OS) and classified according to the ICROP; Stage 1: demarcation line, stage 2: ridge, stage 3: extraretinal fibrovascular proliferation, stage 4: partial retinal detachment, and stage 5: total retinal detachment [15].

Statistical analysis

Excel sheet was used to collect data, and Social Science Package Statistical (SPSS) version 21 was used for statistical analysis. Categorical variables were described as frequencies and percentages, while Mean and standard deviation (SD) were calculated to be reported as continuous variables. Chi-square and student t-test were applied to assess between categorical and continuous variables, respectively. Furthermore, a p-value less than 0.05 was considered significant.

## Results

Our results showed a Female predominance of 51.4%, the mean age of the pregnancy was 27.18 ± 2.29 weeks, the mean birth weight was 0.8 ± 0.26, and 66.7% of our sample was delivered by Cesarean section (Table 1).

**Table 1:** Baseline characteristics of the patients and its association with ROP (N=37) SD: Standard Deviation, kg: Kilogram, SVD: Spontaneous Vaginal Delivery, CS: Cesarean Section, APGAR: Appearance, Pulse, Grimace, Activity and Respiration, OS: Oculus Sinister, OD: Oculus Dextrus

Variables	Number of patients, n (%)	Mean± SD	OD P-value	OS P-value
Gender	-	-	0.407	0.220
Female	19 (51.4)	-	-	-
Male	18 (48.6)	-	-	-
Age (gestational)	-	27.18 ± 2.29	0.016	0.050
Weight (Kg)	-	0.8± 0.26	0.026	0.118
Mode of delivery	-	-	0.898	0.754
SVD	12 (33.3)	-	-	-
CS	24 (66.7)	-	-	-
1-Minute APGAR Score	-	4.8 ± 2.7	0.484	0.247
5-Minute APGAR Score	-	7 ± 1.9	0.720	0.501

A significant association was found between birth weight and the development of ROP in the right eye (p = 0.026) (Table 1). Another significance was found between the gestational Age and the development of ROP in the same eye (p = 0.016). Other baseline characteristics did not show any significant results in developing ROP.

**Figure 1:**
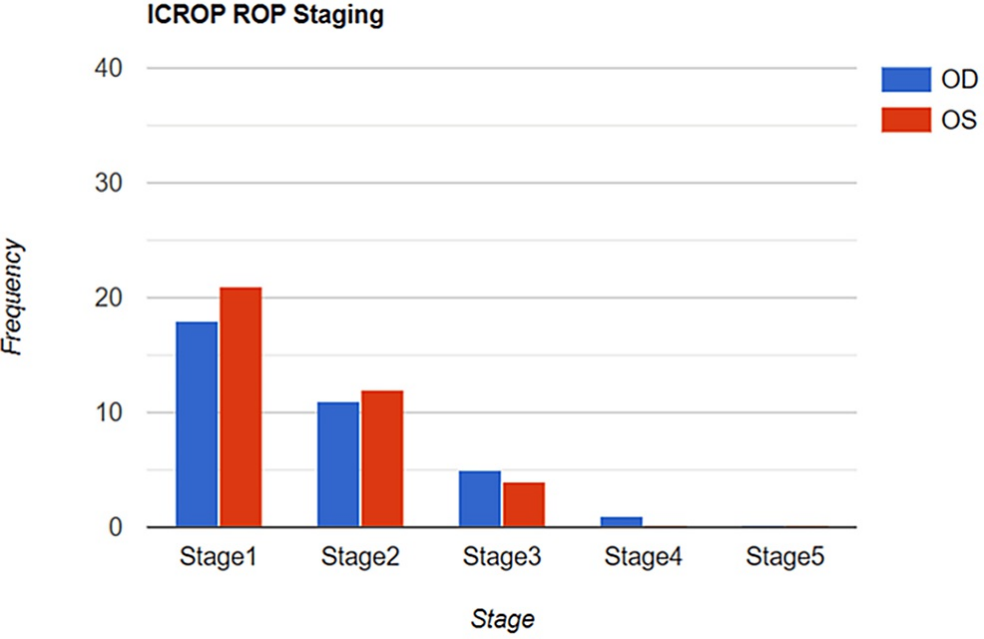
Frequency of ICROP stages in 37 preterms diagnosed with ROP OS: Oculus Sinister, OD: Oculus Dextrus

Both eyes of preterms were classified according to the ICROP for the development of ROP and showed a stage 1 predominance with 18 for OD and 21 for OS, respectively. Stage 2 was recorded for 11 of OD and 12 of OS. 5 OD and 4 OS were recorded for stage 3, and stage 4 was the least with 1 for OD (Figure 1).

The laboratory findings are shown in Table 2. The mean hemoglobin was (15.5 ± 3), the mean leukocyte was found to be (10.8 ± 7.7), and the platelet count was (209± 99). When analyzing the laboratory characteristics, another positive correlation was found in the left eye between the red cell distribution width count and the development of ROP (p = 0.034). No other significant associations were found with the development of ROP in either eye.

**Table 2:** Laboratory characteristics at birth and its association with ROP OS: Oculus Sinister, OD: Oculus Dextrus, SD: Standard Deviation

Laboratory characteristics at birth	Mean± SD	OD P-value	OS P-value
Hemoglobin	15.5 ± 3	0.210	0.088
Hematocrit	46.4 ± 9	0.513	0.194
White Blood Cells	10.8± 7.7	0.167	0.068
Mean Corpuscular Hemoglobin	38.5± 4.5	0.988	0.835
Mean Corpuscular Volume	112 ± 22.9	0.881	0.956
Red Cell Distribution Width	16.9± 2	0.324	0.034
Platelet Count	209± 99	0.374	0.127

## Discussion

This study aims to assess the preterms' initial clinical and laboratory characteristics at the time of birth and identify its correlation with the development of ROP at a tertiary care center in Jeddah, Saudi Arabia. The study analysis showed an inverse relation between neonates' birth weight and the development of ROP in OD (p-value = 0.026), but this was not applicable to the OS. this partially agrees with Lazo et al., which also found low birth weight is a risk factor for ROP [18]. Another retrospective study by Kumaravel et al. revealed that neonates with a birth weight of less than 1,500 grams had higher rates of laser photocoagulation therapy for ROP treatment compared with higher birth weights [20]. This might be because the lower the birthweight, the more likely it is for a neonate to require oxygenation intervention which is an independent risk factor for the development of ROP [21,22]. Thus, we recommend that oxygenation therapy for preterms should be given with caution, and regular monitoring is advised. 

Another significant association was found between gestational age and the severity of ROP in the OD (p-value = 0.016). This finding broadly supports the work of other studies in this area linking the gestational age of newborns with the development and severity of ROP [11,23-25]. A possible explanation for these results may be that the lower the gestational age, the higher risk of incomplete maturation and growth of the body and subsequent arrest in the normal development of the retinal nerve and blood supply. This can be regarded as the driving factor in developing ROP, as it also contributes to our aforementioned result regarding low birth weight. More and more studies from different parts of the world have studied this factor and concluded on its significance. This only reinforces the importance of screening and following up on such neonates with extreme caution.

Contrary to expectations, this study did not find a significant difference between 1- or 5-minute APGAR scores and the development of ROP. This is in contrast to Al Qahtani et al., who found 1-minute APGAR scores to be significantly associated with the development of ROP, but not a 5-minute score [11]. Considering that other variables, such as low gestational age and birth weight, can be detrimental to the neonates' development, is somewhat surprising that APGAR scores did not correlate with ROP. Further studies that investigate the APGAR scores association with ROP need to be undertaken.

Furthermore, a retrospective study by Englert et al. found that anemia was not identified as an independent risk factor for the severity of ROP in preterm infants. However, the number of blood transfusions did affect the severity of ROP in a directly proportional relation. This study goes in parallel with our findings which showed no significance between anemia in preterm neonates at the time of birth and the development of ROP. This reveals that close monitoring of those preterms is better than leaving an anemic child without transfusion [17].

Our study showed no significance between platelet count and the development of ROP in either eye. This goes against a review by Seliniotaki et al., which concluded that platelet deficiency was associated with severe forms of ROP [26]. This conflict in results between our study and others in the literature might be due to the fact that the majority of thrombocytopenic neonates who were included in our study had mild platelet deficiency. Further research is recommended to measure the existence of a specific threshold in which platelet deficiency could lead to ROP progression.

Our study was limited by being a single study location, a small sample size, and poor documentation of data for some patients. A multicenter and multiethnicity study is recommended to understand better the factors related to ROP.

## Conclusions

This research aim was to study and analyze the laboratory and birth characteristics of preterm infants diagnosed with ROP. Birth weight was found to be a significant risk factor in the development of ROP. Furthermore, Gestational age was also found to be significantly associated with ROP severity. As ROP prevalence is increasing every decade, it is important to point out what may contribute to its development and development; these findings help to reinforce the importance of proper screening and follow-up with such neonates. Therefore, we recommend future studies to analyze other aspects of a neonate at the time of birth that could contribute to the development of ROP.
